# Effect of population, collection year, after-ripening and incubation condition on seed germination of *Stipa bungeana*

**DOI:** 10.1038/s41598-017-14267-2

**Published:** 2017-10-24

**Authors:** Rui Zhang, J. M. Baskin, C. C. Baskin, Qing Mo, Lijun Chen, Xiaowen Hu, Yanrong Wang

**Affiliations:** 1 State Key Laboratory of Grassland Agro-ecosystems, College of Pastoral Agriculture Science and Technology, Lanzhou University, Lanzhou, 730020 China; 2 Department of Biology, University of Kentucky, Lexington, KY 40506 USA; 3 Department of Plant and Soil Sciences, University of Kentucky, Lexington, KY 40546 USA

## Abstract

Knowledge of the germination behavior of different populations of a species can be useful in the selection of appropriate seed sources for restoration. The aim of this study was to test the effect of seed population, collection year, after-ripening and incubation conditions on seed dormancy and germination of *Stipa bungeana*, a perennial grass used for revegetation of degraded grasslands on the Loess Plateau, China. Fresh *S. bungeana* seeds were collected from eight locally-adapted populations in 2015 and 2016. Dormancy and germination characteristics of fresh and 6-month-old dry-stored seeds were determined by incubating them over a range of alternating temperature regimes in light. Effect of water stress on germination was tested for fresh and 6-month-old dry-stored seeds. Seed dormancy and germination of *S. bungeana* differed with population and collection year. Six months of dry storage broke seed dormancy, broadened the temperature range for germination and increased among-population differences in germination percentage. The rank order of germination was not consistent in all germination tests, and it varied among populations. Thus, studies on comparing seed dormancy and germination among populations must consider year of collection, seed dormancy states and germination test conditions when selecting seeds for grassland restoration and management.

## Introduction

In ecological restoration, proper sourcing of seeds is a primary consideration for improving planting success and ensuring that new populations are functional, self-sustaining and resilient to environmental challenges^1^. Local populations are often recommended as the seed source in ecological restoration since they frequently are adapted to the local environment and thus facilitate vegetation establishment^2–4^. However, some studies have reported that non-local populations have higher fitness than local populations^5–10^. Seed dormancy and germination are crucial stages in the life cycle of plants, and thus they play an important role in conservation and revegetation of degraded sites^11,12^. Previous studies have shown that seed dormancy and germination can vary greatly among populations^13^, and these early life history traits have been found to affect seedling establishment and plant fitness^13,14^. Thus, information on germination characteristics of seeds from different populations is necessary for selection of the most suitable seed population in ecological restoration.

Seed dormancy and germination traits have been used to select the proper seed source in restoration practice^10,15,16^. A general hypothesis underpinning this kind of research is that seeds with similar dormancy and germination behavior will result in similar performance in later life history stages. This may be true since population differences have been found play a key role in controlling seed dormancy and germination^13,17^. For instance, in *Brassica oleracea* variation in germination percentages and rates among populations are largely attributed to genetics^18^. However, in addition to genetic effects, seed dormancy and germination are strongly dependent on maternal environment during seed development^10,19–21^. For example, warm, dry growing conditions of the mother plant decreased the intensity of non-deep physiological dormancy of *Alopecurus myosuroides* seeds compared to cool, wet conditions^22^. Regardless of latitude of collection site, *Arabidopsis thaliana* plants grown at a low (10 °C) temperature produced seeds that were more dormant than those grown at a high (22 °C) temperature^23^. In particular, seed germination of a species collected at different sites may vary between years^24–26^. Thus, it is of interest to know if between-year variation in dormancy and germination override the effect of population.

Seed dormancy state also is influenced by after-ripening^13,26^ and cold stratification^27,28^, which can differ among populations. For example, between-population differences in germination of *Bromus tectorum* L. are less evident for after-ripened than for recently-harvest seeds^26^. Milberg and Andersson (1998) found that population differences in dormancy after cold stratification and/or after-ripening varied with species^27^. Further, the speed of seed after-ripening can vary among populations^13^, which implies that after-ripening can decrease or increase differences among populations. Thus, we need to know the role of after-ripening in the variation of dormancy and germination among populations.

Moreover, the interactive effects of population and germination conditions (e.g. moisture and temperature) have been extensively reported^13,29–31^. For instance, seeds of *Pinus brutia* differ among populations in their sensitivity to moisture stress^30^, and those of *Acacia lebbek* vary in temperature requirements for germination^29^. Further, seed germination requirement usually is synchronized with environmental conditions in the habitat that are favorable for seedling establishment^13,32^. Thus, understanding the variation of seed germination responses to a wide range of environmental conditions is helpful in choosing appropriate seed lots for ecological restoration.

*Stipa bungeana* is a dominant perennial (tussock) grass widely distributed on the Loess Plateau and other areas of western China^33^, but the quality of most of the grassland in this region is declining due to decreasing dominance of this species^34^. Thus, it is necessary to select proper seed sources of *S. bungeana* for grassland restoration since it is a key species for revegetation of degraded land on the Loess Plateau, due to its importance in protecting the soil from erosion and reducing water loss by runoff^33^. Seeds of *S. bungeana* have non-deep physiological dormancy at the time of dispersal in late June and only a small proportion of them have potential to form a persistent seed bank. Seedling emergence mainly occurs from July to September in the field since most of seeds undergo after-ripening during summer^33,35^. Jing *et al*. (2013) found a high level of genetic diversity among populations of *S. bungeana* from Shaanxi, Inner Mongolia, Ningxia and Gansu provinces of China^36^. Thus, we hypothesized that differences in seed dormancy and germination among populations are affected by seed collection year, seed dormancy state and other factors. To test this hypothesis, we sought answers to the following questions. 1) Do seed dormancy and germination of *S. bungeana* differ among populations? 2) Do among-populations differences in dormancy/germination vary with year? 3) Can dry storage (after-ripening) of seeds level out among-populations differences in dormancy and germination?

## Results

### Main effects

**Figure 1:**
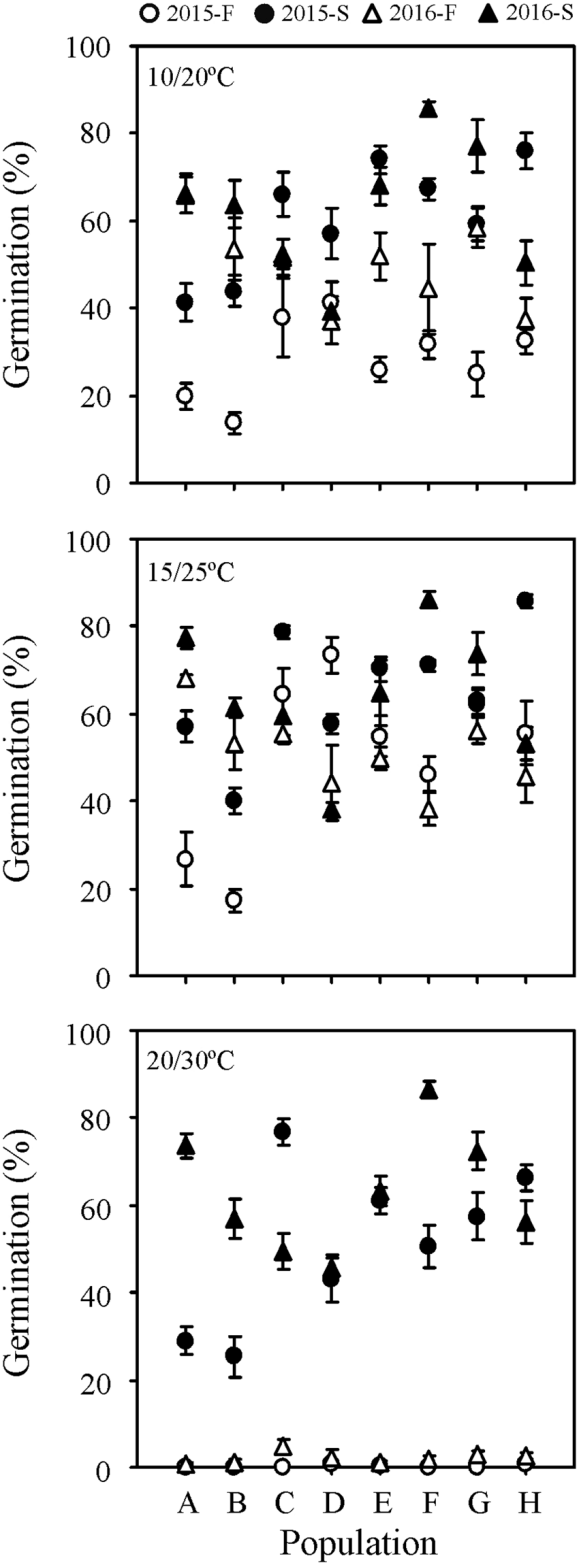
Germination percentages (mean ± se, n = 6) of fresh and 6-month-old dry stored *Stipa bungeana* seeds from eight populations at three temperatures regimes in light (12 h/12 h) in 2015 and 2016. 2015-F, fresh seeds collected in 2015; 2015-S, 6-month-dry stored seeds collected in 2015; 2016-F, fresh seeds collected in 2016; 2016-S, 6-month-dry stored seeds collected in 2016.

**Figure 2:**
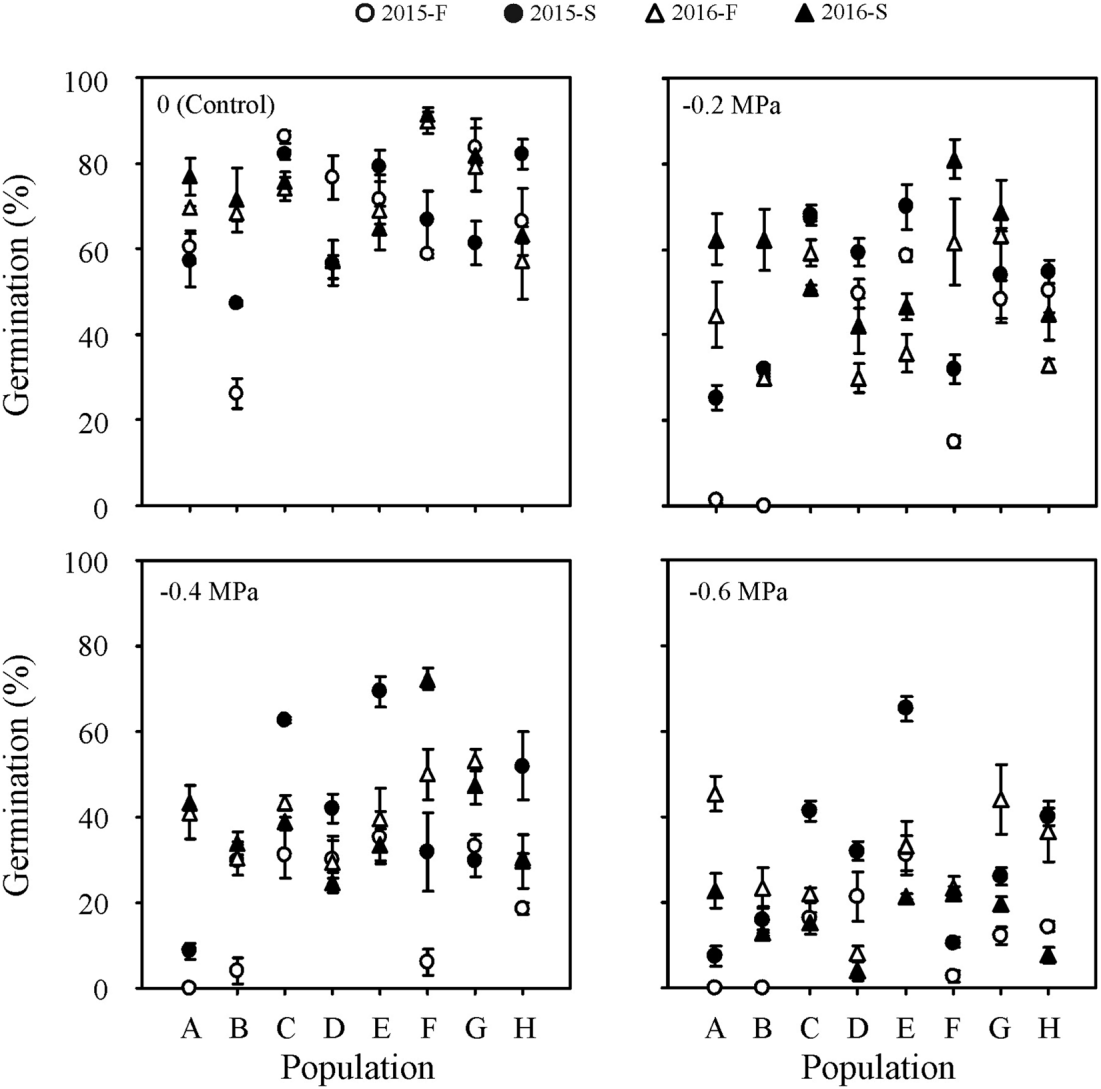
Effect of water potential on germination percentages (mean ± se, n = 4) of fresh and 6-month-old dry stored *Stipa bungeana* seeds from eight populations in 2015 and 2016 at 20 °C in continuous dark condition. 2015-F, fresh seeds collected in 2015; 2015-S, 6-month-dry stored seeds collected in 2015; 2016-F, fresh seeds collected in 2016; 2016-S, 6-month-dry stored seeds collected in 2016.

**Figure 3:**
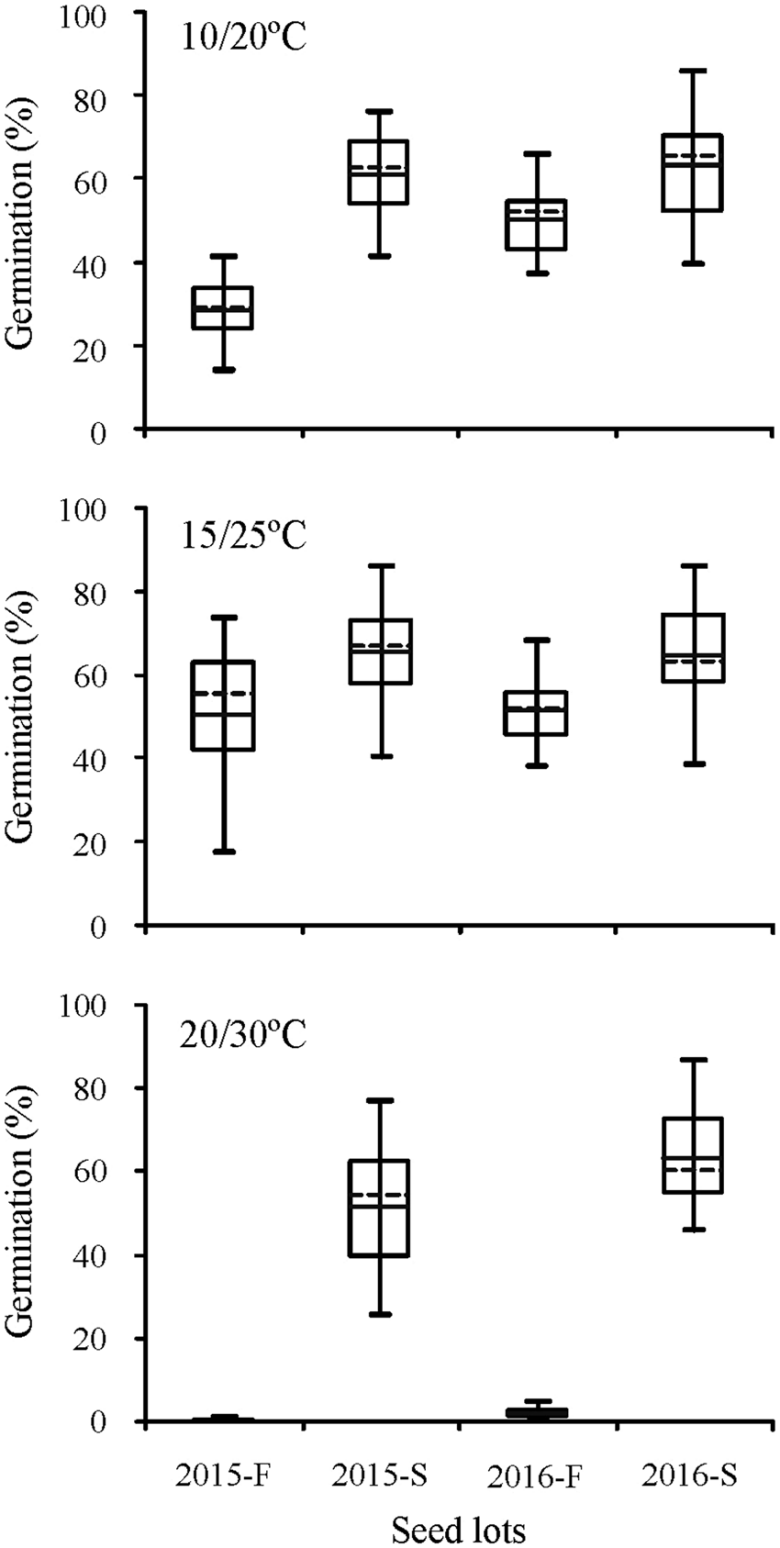
Germination percentage (mean ± se, n = 6) of fresh and 6-month-old dry stored *Stipa bungeana* seeds from eight populations in 2015 and 2016 at three temperatures. The mean is indicated by a horizontal dotted line and the 25th, 50th and 75th percentiles by solid horizontal lines. Solid horizontal lines outside the box plots display the minimum value and maximum value. 2015-F, fresh seeds collected in 2015; 2015-S, 6-month-dry stored seeds collected in 2015; 2016-F, fresh seeds collected in 2016; 2016-S, 6-month-dry stored seeds collected in 2016.

**Figure 4:**
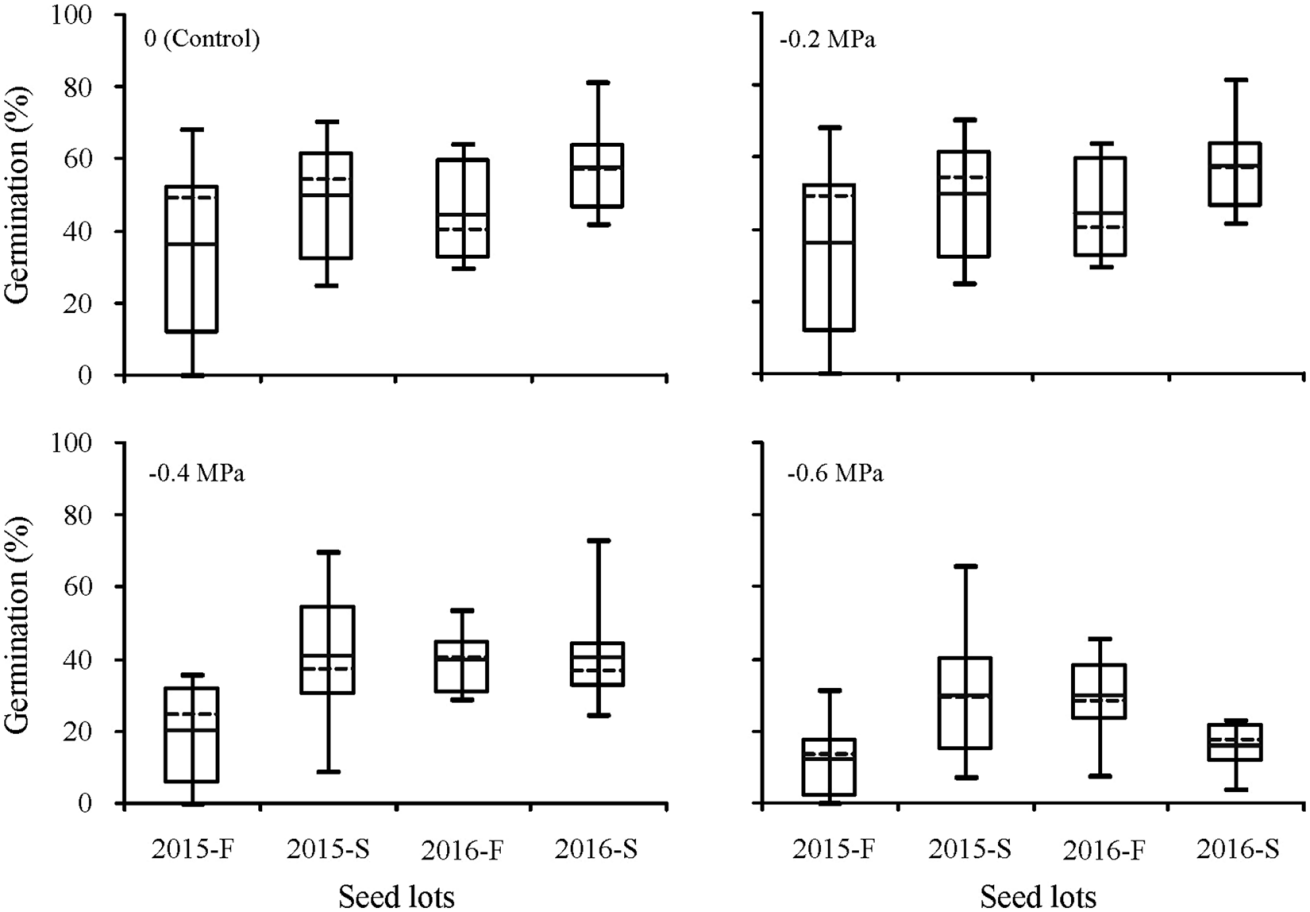
Germination percentage (mean ± se, n=4) of fresh and 6-month-old dry stored *Stipa bungeana* seeds from eight populations in 2015 and 2016 under four water potentials. The mean is indicated by a horizontal dotted line and the 25th, 50th and 75th percentiles by solid horizontal lines. Solid horizontal lines outside the box plots display the minimum value and maximum value. 2015-F, fresh seeds collected in 2015; 2015-S, 6-month-dry stored seeds collected in 2015; 2016-F, fresh seeds collected in 2016; 2016-S, 6-month-dry stored seeds collected in 2016.

Year, storage, temperature, population and most of their interactions had significant effects on seed germination of *Stipa bungeana* (Table S1), as did storage, water potential, population and all of their interactions except for storage × water potential × population (Table S2). Seed germination percentage of *S. bungeana* varied with population, year of seed collection, seed dormancy state (fresh/stored) and germination condition (temperature/water stress) (Figs 1,2). Germination percentage at different temperatures and levels of water stress increased after seeds had been stored dry for 6 months, and it varied with population, year and germination condition (Figs 3,4).

### Population

Differences in seed dormancy and germination among populations were significant, and there was no consistent pattern in the ranking by population. *S. bungeana* seeds had non-deep physiological dormancy, which varied in its depth depending on population (Figs 1,2). Germination percentage of fresh seeds in 2015 ranged from 13.7% to 41.2% at 10/20 °C, 17.4% to 73.4% at 15/25 °C, and 0% to 0.7% at 20/30 °C. There was wide variation in germination at different temperatures except for fresh seeds at 20/30 °C, where few or no seeds germinated (Fig. 3). Difference in germination percentage among populations varied with seed dormancy state, year of seed collection and germination condition.

### Storage

**Figure 5:**
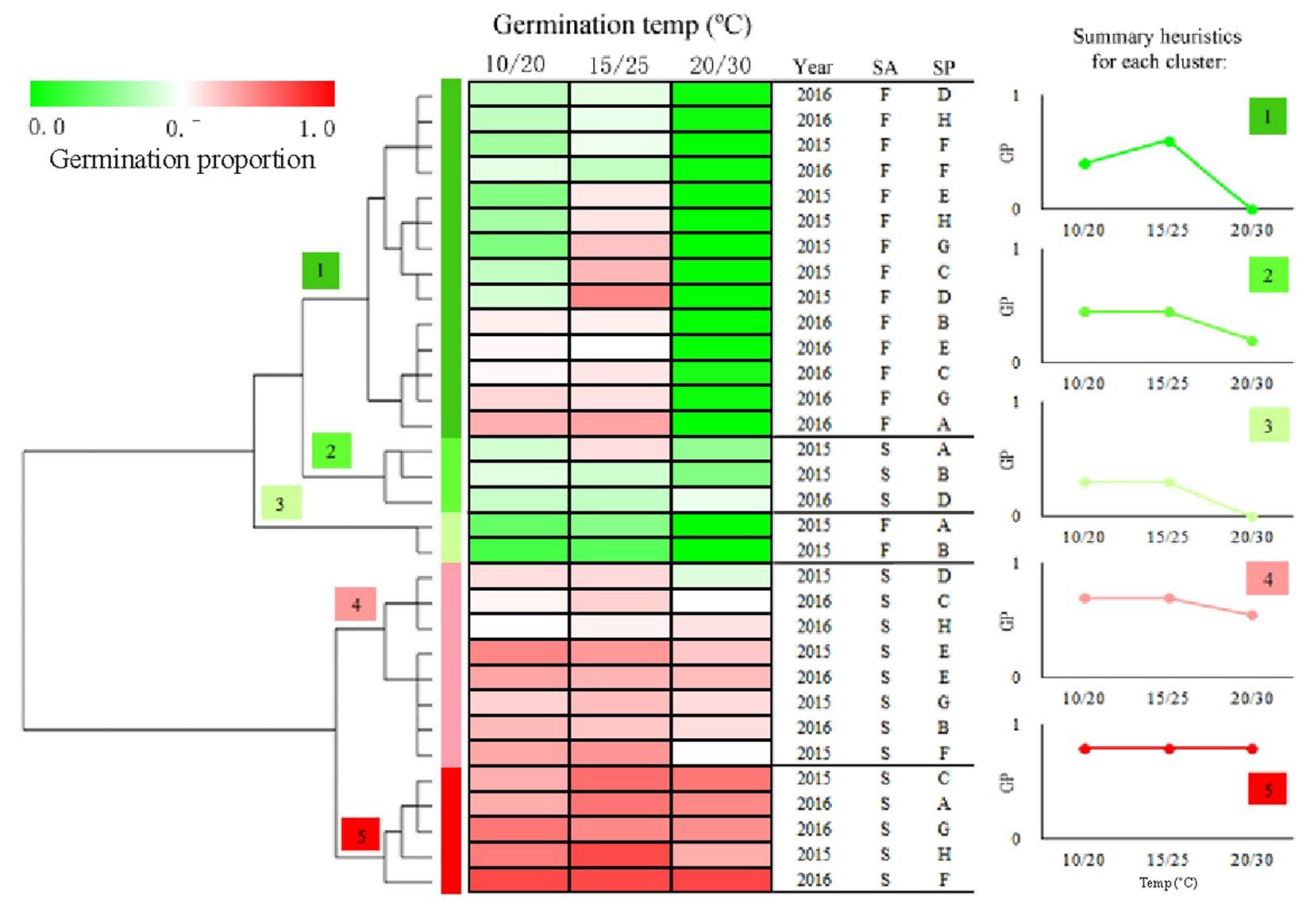
Cluster dendrogram and heat map of similarity of *Stipa bungeana* germination envelopes at three temperatures for all combinations of seed collection year (Year), seed dormancy state (SA) and seed population (SP). The color scale indicates germination percentage: deeper green color shades indicate lower germination percentage, more intense red color higher germination percentage and white germination percentage 50%.

Six months of dry storage broadened the temperature range for germination (Fig. 1) and increased seed tolerance to water stress (Fig. 2). The mean germination percentage of *S. bungeana* seeds from the eight populations increased for seeds that had been stored dry for 6 months (Figs 3,4). Clusters 4 and 5 (stored seeds) had high germination percentages, especially cluster 5, whereas little germination occurred in clusters 1–3 (fresh seeds), indicating that most of the dormancy was lost during 6 months of dry storage (Fig. 5). The amount of variation in germination among populations was greater after 6 months of dry storage than it was for fresh seeds, except for seeds collected in 2015 and incubated at 15/25 °C (Fig. 3), seeds collected in 2015 incubated under 0 (control) and −0.2 MPa and seeds collected in 2016 incubated under −0.6 MPa (Fig. 4).

### Year

Germination response patterns were not consistent from year to year, and from the same population they increased or decreased across years (Figs 3,4). For fresh seeds incubated at 15/25 °C, germination percentages from populations A and B increased from 2015 to 2016, while they decreased for the other populations (Fig. 1). For 6-month dry stored seeds incubated at 15/25 °C, germination percentages of seeds from populations A, B, F and G increased from 2015 to 2016, while they decreased for the other populations. For 6-month dry stored seeds, germination percentage of seeds from population A exhibited the most across-year differences under most conditions, whereas that of seeds from population E exhibited the least difference (Fig. 1). Moreover, the pattern of between-year variation in germination was not consistent under different water potentials (Fig. 4). When incubated under −0.6 MPa, variation in germination between years was greater for fresh seeds than that for those stored dry for 6 months.

### Germination conditions

**Figure 6:**
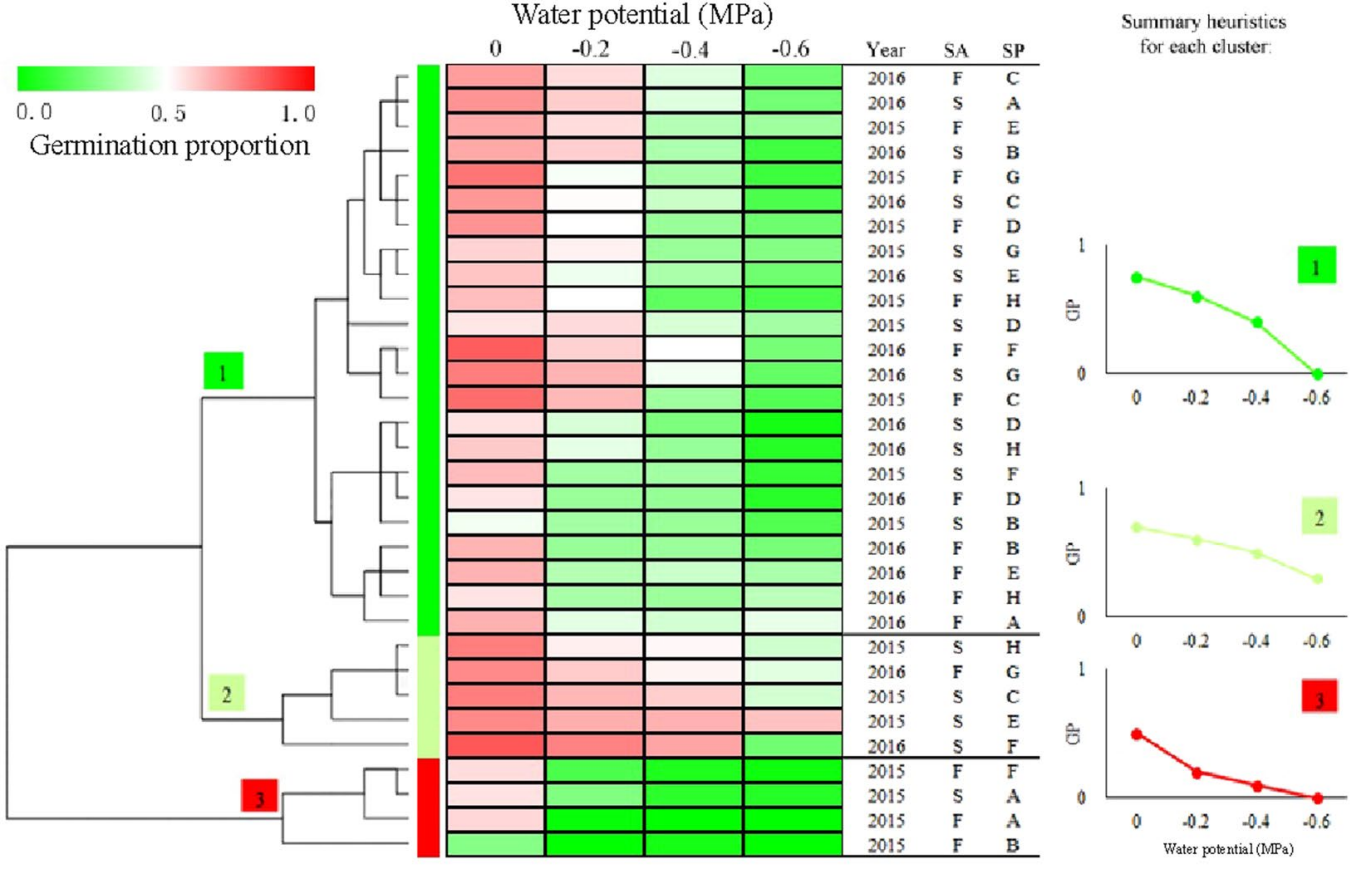
Cluster dendrogram and heat map of similarity of *Stipa bungeana* germination envelopes under four water potentials for all combination of seeds collection year (Year), seed dormancy states (SA) and seed population (SP). The color scale indicates germination percentage: deeper green color shades indicates lower germination percentage, more intense red color higher germination percentage and white germination percentage 50%.

Differences in germination among populations also varied with germination conditions (Figs 1,2). There was wide variation among populations in germination response to temperature and water stress, except at 20/30 °C, where no fresh seeds germinated (Fig. 1). Germination percentage decreased with decreasing water potential for all populations, and sensitivity to water stress varied with population, seed dormancy state and year (Fig. 2). Germination responses to temperature and water stress changed with year, seed dormancy state and population, and different combinations of these three factors produced similar germination envelopes (Figs 5,6).

## Discussion

**Figure 7:**
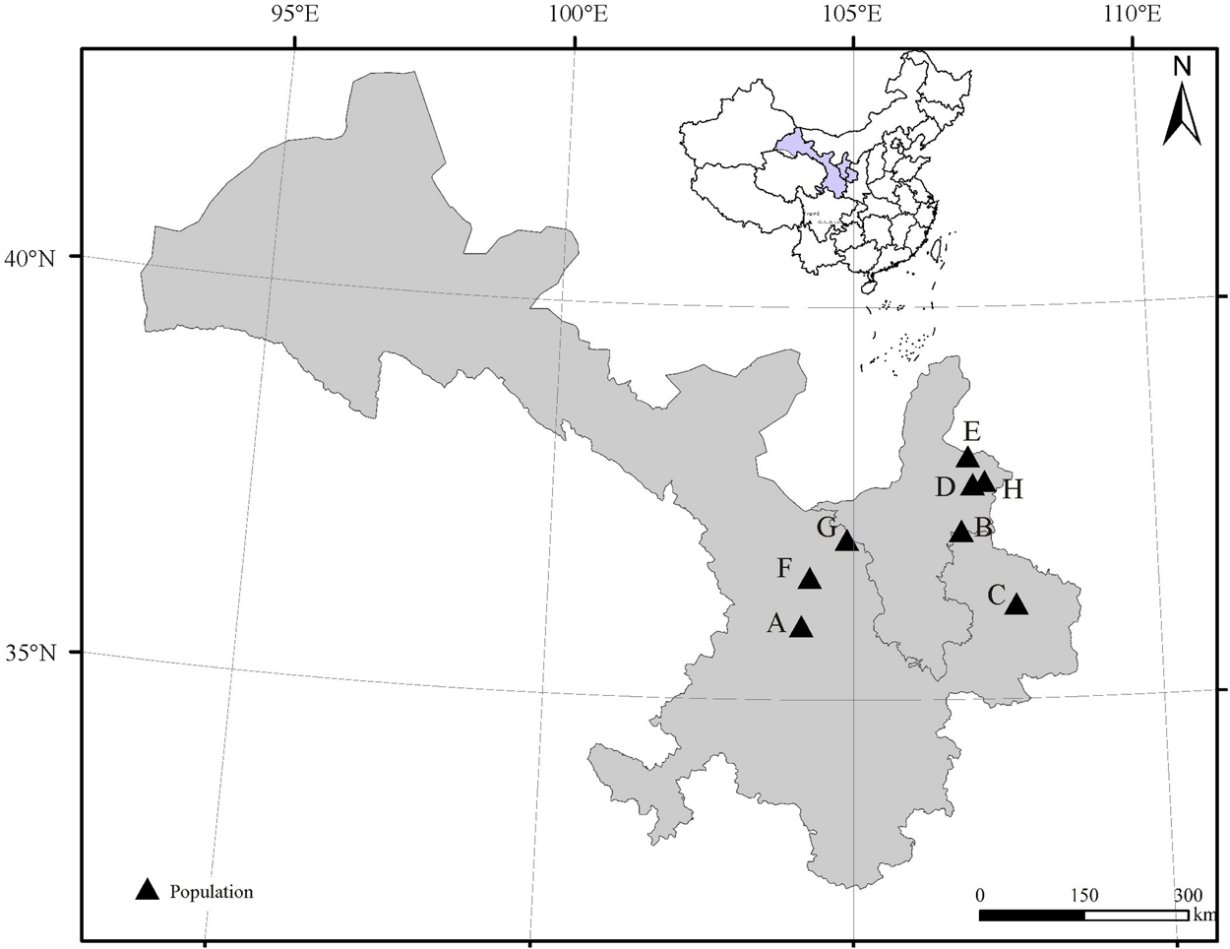
Locations of seed collection sites on the Loess Plateau, China. A-H, collection sites. The map was created using ArcView GIS (version 3.2, http://www.resources.esri.com). The base map was obtained by public DIVA-GIS (URL: http://www.diva-gis.org/gdata).

Our study clearly shows that seed dormancy and germination of *S. bungeana* differ significantly among populations. Gene flow among *S. bungeana* populations is limited on the Losses Plateau, and a high level of genetic differentiation exists among populations^36^. In the present study, the distance between any two of the seed collection sites is >10 km (Fig. 7), and there likely are genetic differences among the eight populations. Thus, genetic differences may contribute to the variation we found for seed dormancy and germination.

In the study by Jing *et al*. (2013), genetic distance was not significantly correlated with geographical distances in *S. bungeana* because of genetic drift. Although geographic distances between populations D, E and H in the present study are closer than the geographic distance for any of the other populations, germination percentage of 2015 fresh seeds from population D at 15/25 °C was significantly higher than that at other two populations. In contrast, germination percentages of fresh seeds from populations A and B, which are far apart, were similar. The results indicate that differences in germination among populations had no relationship with geographic distances. Germination percentage of fresh seeds at 15/25 °C from populations D and H differed significantly although they are within the same city (Yanchi, Ningxia Province). A possible explanation for this result is that germination differences of the two populations are due to genetic differentiation^18^. Another possible explanation for the differences is that they are due to maternal environment since environmental conditions between populations that are geographically close to each other can either be similar or very different^37^.

Seed germination may be closely related to factors of the maternal environment, such as competition^38^, day length^39^, light quality^40^, mineral nutrition^41^, soil moisture^22^, temperature^42^ and so on^13^. Since the maternal environment in the field consists of various factors, it is hard to demonstrate which one(s) has(have) an effect on germination characteristics of the seeds^43,44^. In the present study, we considered the factors geographic location and climate (mean monthly temperature and monthly total rainfall) and found that there has no relationship between geographic location and germination percentage at any test condition (Tables S3A,B). However, mean monthly temperature of seed maturation had a significant effect on germination of fresh seeds at 10/20 °C (Table S4A), indicating that seeds of *S. bungeana* matured in cool conditions were more dormant than those matured in warm conditions, which is consistent with a study on *Alopecurus myosuroides* ^22^. Indeed, we found that seed collection year, storage (seed dormancy state)^13,26–28^, germination temperature and moisture stress^29–31^ interactively affected germination of seeds from different populations.

### Year

Germination percentage varied for seeds collected at the same site between 1971 and 1973 in *Artemisia tridentata* ^45^, between 1969 and 1971 in *Sitanion hystrix* var. *hystrix* ^43^ and between 2001 and 2002 in *Minuartia recurva* subsp. *bigerrensis* and *Jasione crispa* subsp. *centralis* ^46^. Similarly, germination percentages of *S. bungeana* seeds collected in 2015 differed from those collected at the same sites in 2016. Variation in dormancy and germination between years depends on the species^45^, populations^47^ and amount of year to year environmental changes^46^. Germination of *Artemisia tridentata* seeds differed significantly in year-to-year variation between 1971 and 1973, while seeds of *A. wyomingensis* and *A. vaseyana* did not^45^.

The maternal environment (soil moisture/water stress, temperature, different years) during the time of seed development could cause differences in seed dormancy and germination^22,42^. The intensity of dormancy in seeds of *Alopecurus myosuroides* was higher for seeds from plants growing in cool, wet conditions than for those growing in warm, dry conditions^22^. However, seeds from plants of *Arachis hypogaea* ^48^ and *Cenchrus ciliaris* ^49^ growing at low soil moisture were more dormant than those grown at high soil moisture. Seeds of *Arabidopsis thaliana* produced at high temperature had higher germination percentages than those produced at low temperatures^50,51^. In contrast, seeds of *Lactuca sativa* produced at high temperature were more dormant than those produced at low temperature^52^. Germination percentages of *Ifloga spicata*, *Rumex pictus* and *Senecio glaucus* seeds were higher for those produced in dry than in wet years^53^. Likewise, germination percentages of seeds of *Artemisia rhodantha* produced in warm years were higher than those produced in cool years^54^.

Germination percentage of fresh seeds of population D incubated at 15/25 °C was 73.4% in 2015 and 44.2% in 2016, when the monthly total rainfall during the seed maturation period was 11.8 mm and 20.3 mm, respectively. Germination percentage of seeds from population A was 26.8% in 2015 and 68.0% in 2016, when the monthly total rainfall of seed maturation period was 33.7 mm and 62.1 mm, respectively. Germination percentage of seeds from population E did not differ significantly between 2015 and 2016, while the monthly total rainfall of seed maturation period was 8.6 mm and 9.8 mm, respectively. As with the rainfall during the seed maturation period, germination response to the mean monthly temperature in this period also was not consistent. Germination percentages of fresh seeds of population A incubated at 15/25 °C increased significantly from 26.7% to 68.0% as mean temperature increased from 17.5 °C to 22.1 °C, and those of fresh seeds of population B increased from 17.0% to 53.0% as mean temperature increased from 20.4 °C to 23.6 °C. Germination percentages of fresh seeds from the other populations incubated at 15/25 °C decreased with an increase in temperature. To sum up, there was no consistent pattern between germination response and rainfall and temperature across years during seed development^13^. Between-year differences in germination of *S. bungeana* seeds could not be easily accounted for by variation in weather conditions during seed maturation. That is, it is hard to demonstrate which factor(s) of the maternal field environment influence germination characteristics of the seeds^43,44^. In any case, the effect of year as well as other factors of maternal environment should be taken into consideration in estimating variation in germination among population. However, it is worth noting that the results are based on data for only 2 years, and further study involving multiple years may provide a more robust estimation of the effect of collection year on seed germination.

### Storage

Germination percentage of seeds with physiological dormancy such as those of *S. bungeana* ^33^ typically increases with after-ripening^13^. Six months of dry storage broke seed dormancy and broadened the temperature range for germination, especially at the high temperature regime (20/30 °C). The speed of after-ripening and dormancy status can vary, depending on environmental conditions during seed maturation, storage and germination conditions^13,55^. Moreover, population differences in dormancy and germination may be disappear in some species (*Chenopodium album*, *Anchusa arvense*, *Chenopodium suecicum*, *Stellaria media*) after cold stratification, while in other species (*Galeopsis speciosa*, *Lamium hybridum*, *Buglossoides arvensis*, *Sonchus asper*) population differences may become apparent after stratification^13,27^. The effect of after-ripening on germination in *S. bungeana* varied with population. For instance, germination percentage of seeds from population A incubated in light at 15/25 °C in 2015 increased from 26.8% in fresh seeds to 57.0% in 6-month-old dry stored seeds. In contract, germination percentage ranged from 62.4% to 63.0% for seeds of population G. Further, cluster results (Fig. 5) clearly showed that germination characteristics were similar for fresh seeds from populations D and H in 2016 and that the two populations were clustered in different groups (population D in group 2 and population H in group 4) after 6 months dry storage.

Beckstead *et al*. (2011) reported that between-population differences in germination of *Bromus tectorum* seeds were greatest for those recently harvested and least for after-ripened seeds, since after-ripened seeds became nondormant and germinated more uniformly than freshly matured seeds^26^. However, variation in germination percentage among populations of *S. bungeana* had increased after 6 months dry storage, except for seeds collected in 2015 incubated at 15/25 °C. Differences between the results of the Beckstead *et al*. (2011) and our study may be due to species differences. It is worth noting that population differences in germination of *S. bungeana* seeds incubated at 20/30 °C occurred after they were dry stored for 6 months, while no difference was found for fresh seeds, i.e. no seeds germinated. Therefore, seed dormancy state (storage) should be taken into account in comparing the differences in germination among populations.

### Germination conditions

Seed germination is strongly influenced by variation in temperature, water stress and light/dark requirements^13^, and comparison of the germination characteristics of a species among populations should take these factors into consideration. For example, germination of fresh seeds from the eight seed provenances did not differ in germination at 20/30 °C since basically no germination occurred. This temperature regime is similar to that seeds currently experience after dispersal in the late June, which indicates that *S. bungeana* seeds are probably “programmed” to germinate after the summer season has passed. Hu *et al*. (2013) also showed that temperatures >25 °C significantly reduced the speed of germination and restricted seedling recruitment in the field in mid-summer^33^. However, germination of fresh seeds in our study differed at 10/20 °C and 15/25 °C, indicating that possible population differences can be ignored when no seeds germinate at 20/30 °C. In addition, seeds from all eight populations differed in their germination response to different levels of water potential (Fig. 6). Ranking of populations according to germination percentage was not consistent across all test conditions, and rank order of populations changed with germination conditions. Thus, differences/similarities in germination among populations should be confirmed under various conditions. Moreover, comparison of populations based on germination across various conditions will indicate an adaptation to specific habitat conditions^10^.

## Materials and Methods

### Seed collection

In late June 2015 and 2016, freshly matured dispersal units of *S. bungeana* were collected from the same eight natural populations on the Loess Plateau in northern China (Figure 7, Table 1). Data for mean monthly temperature and monthly total rainfall (Table 2) during the seed maturation period (June) for collection sites were obtained from nearby weather stations. The dispersal unit (hereafter seed) of *S. bungeana* is a caryopsis (hereafter seed) tightly enclosed by the palea and lemma^35^, and the lemma has a long awn that can anchor and effectively drill the seed into the ground^56^. Seeds were collected from several hundred plants at each of the eight collection sites and taken to the laboratory. The awns were removed by hand, and then the seeds were cleaned, dried at room temperature for 1 week (RH 20–35%, 18–25 °C) and stored at 4 °C until used in experiments. Except for seeds allowed to after-ripen for 6 months before they were tested for germination, germination tests were conducted within 2 weeks after seed collection.

**Table 1:** Geographical locations and altitudes of the eight populations of *Stipa bungeana* from which seeds were collected for the present study.

Population	Collection site	Longitude (E)	Latitude (N)	Altitude (m)
A	Yuzhong	104°10′21″	35°57′19″	1704
B	Huanxian	106°46′59″	37°08′58″	1499
C	Qingcheng	107°39′18″	36°13′26″	1121
D	Yanchi_1_	106°58′59″	37°44′23″	1333
E	Qingtongxia	106°54′10″	38°05′48″	1346
F	Jingyuan_1_	104°16′43″	36°33′34″	1702
G	Jingyuan_2_	104°53′34″	37°02′43″	1563
H	Yanchi_2_	107°11′06″	37°46′30″	1502

**Table 2:** Mean monthly temperature and monthly total rainfall in June (seed maturation period) for eight study populations of *Stipa bungeana* seeds in 2015 and 2016.

Populations	Temperature		Rainfall	
	2015	2016	2015	2016
A	17.5	22.1	33.7	62.1
B	20.4	23.7	38.1	80.4
C	—^a^	—	—	—
D	20.9	21.4	11.8	20.3
E	21.4	21.7	8.6	9.8
F	21.4	22.5	27.7	18.1
G	21.4	22.5	27.7	18.1
H	18.4	19.5	22.8	21

^a^Data not available.

### Germination tests

Germination of *S. bungeana* seeds collected from the eight natural populations in 2015 and in 2016 was tested at 10/20, 15/25 and 20/30 °C in light (12 h/12 h, white fluorescent tubes, photon flux density of about 60 µmol m^−2^ s^−1^, 400–700 nm). The three temperature regimes are those at which seeds are considered to germinate at in the field. For each treatment, six replicates of 50 seeds were placed in 9-cm-diameter Petri dishes on two sheets of filter paper moistened with 5 ml distilled water or test solution. Germination was monitored daily for 14 days, and a seed was counted as germinated when the radicle was visible.

To determine the effect of dry storage (after-ripen) on seed dormancy break and germination in 2015 and 2016, fresh seeds from each of the eight populations were placed in a paper bag and stored in darkness at 20 °C (RH, 20–35%) for 6 months. After dry storage, germination was tested at 10/20, 15/25 and 20/30 °C in light. There were six replicates of 50 seeds and germination was monitored daily for 14 days as described above.

The effect of water potential was tested on seed germination of fresh and 6-month-dry stored seeds collected from eight populations in 2015 and 2016. Four replicates of 50 seeds were placed in Petri dishes on two layers of filter paper moistened with distilled water (0 MPa) or polyethylene glycol 6000 solution (PEG; −0.2, −0.4 and −0.6 MPa) at 20 °C in darkness for 14 days and checked for germination as described above. The PEG solutions were prepared according to Michel and Kaufmann (1973)^57^. Filter papers and solutions were renewed every 48 h to keep the water potential nearly the same during the germination period.

### Statistical analysis

Four generalized linear mixed effect models (GLMMs) for binary data with binomial error distribution were used in GenStat 18. Collection year, storage (seed dormancy state), temperature/water potential and population were used as fixed effects, while replicates were included as random effects in each model. A cluster analysis was used to examine similarities in germination behavior of 32 combinations of three factors [2 years (Year) × 2 dormancy states (SA) × 8 seed populations (SP)]. All figures were created with Excel 2007.

## Electronic supplementary material

Supplementary Information
