# Supplementary material for: Effect of population, collection year, after-ripening and incubation condition on seed germination of Stipa bungeana
Source: Sci Rep. 2017 Oct 24;7:13893. doi: 10.1038/s41598-017-14267-2 (PMC5655671; doi:10.1038/s41598-017-14267-2)
Supplement: Supplementary file 1 — Supplementary Information [file 41598_2017_14267_MOESM1_ESM.pdf]

Supplementary Information

Effect of population, collection year, after-ripening and incubation conditions on seed germination of *Stipa bungeana*

Rui Zhang[1], J. M. Baskin[2], C. C. Baskin[2, 3], Qing Mo[1], Lijun Chen[1], Xiaowen Hu[1]*, Yanrong Wang[1]*

[1]State Key Laboratory of Grassland Agro-ecosystems, China, College of Pastoral Agriculture Science and Technology, Lanzhou University, Lanzhou, 730020, China;

[2]Department of Biology, University of Kentucky, Lexington, KY 40506, USA;

[3]Department of Plant and Soil Sciences, University of Kentucky, Lexington, KY 40546, USA.

Running head title: seed germination of *Stipa bungeana*

*Author for correspondence: XiaoWen Hu, YanRong Wang

Address: College of Pastoral Agriculture Science and Technology, Lanzhou University, No.768 Jiayuguan West Road, Chengguan District, Lanzhou, 730020, China

Fax: 86-0931-8914043

Tel: 86-13519646178

Email: huxw@lzu.edu.cn; yrwang@lzu.edu.cn

Supplementary Information

Table S1 Results of the generalized linear mixed effects models (GLMMs) on the effect of year (*Y*), storage (seed dormancy states) (*S*), temperature (*T*), population (*P*) and their interactions on seed germination percentage of *Stipa bungeana*.

	Wald statistic	n.d.f.	F statistic	F-pr
Year (*Y*)	16.87	1	16.87	<0.001
Storage (*S*)	267.03	1	267.03	<0.001
Temperature (*T*)	118.31	2	59.15	<0.001
Population (*P*)	128.73	7	18.39	<0.001
$Y \times S$	4.27	1	4.27	0.039
$Y \times T$	27.96	2	13.98	<0.001
$S \times T$	309.22	2	154.61	<0.001
$Y \times P$	341.03	7	48.72	<0.001
$S \times P$	98.03	7	14	<0.001
$T \times P$	23.35	14	1.67	0.059
$Y \times S \times T$	18.28	2	9.14	<0.001
$Y \times S \times P$	66.77	7	9.54	<0.001
$Y \times T \times P$	21.88	14	1.56	0.086
$S \times T \times P$	25.06	14	1.79	0.037
$Y \times S \times T \times P$	6.41	14	0.46	0.954

Table S2 Results of the generalized linear mixed effects models (GLMMs) on the effect of year (*Y*), storage (seed dormancy states) (*S*), water potential (*W*), population (*P*) and their interactions on seed germination percentage of *Stipa bungeana*.

	Wald statistic	d.f.	Wald/d.f.	Chi pr
Year (Y)	0.11	1	0.11	0.737
Storage (S)	22.28	1	22.28	<0.001
Water potential (W)	920.22	3	306.74	<0.001
Population (P)	158.16	7	22.59	<0.001
Y ×S	34.02	1	34.02	<0.001
Y ×W	24.57	3	8.19	<0.001
S ×W	13.64	3	4.55	0.003
Y ×S	485.77	7	69.4	<0.001
S ×P	54.9	7	7.84	<0.001
W ×P	139.41	21	6.64	<0.001
Y ×S ×W	157.74	3	52.58	<0.001
Y ×S ×P	29.03	7	4.15	<0.001
Y ×W ×P	75	21	3.57	<0.001
S ×W ×P	31.93	21	1.52	0.06
Y ×S ×W ×P	37.08	21	1.77	0.016

Table S3A Correlations between geographic location and germination percentages of fresh and stored seeds of *Stipa bungeana* from eight natural populations incubated at three temperature regimes.

		Germination percentage (%)					
		10/20 ℃		15/25 ℃		20/30 ℃	
		Fresh	Stored	Fresh	Stored	Fresh	Stored
Longitude	R	-0.054	-0.229	0.159	-0.307	0.273	-0.138
	P	0.843	0.393	0.555	0.248	0.307	0.609
Latitude	R	-0.146	0.055	0.076	-0.245	0.022	-0.066
	P	0.589	0.838	0.780	0.360	0.934	0.809
Altitude	R	-0.066	0.185	-0.332	0.210	-0.333	0.031
	P	0.809	0.492	0.209	0.436	0.207	0.911

Table S3B Correlations between geographic location and germination percentages of fresh and stored seeds of *Stipa bungeana* from eight natural populations incubated at four water potentials.

		Germination percentage (%)							
		CK		-0.2		-0.4		-0.6	
		Fresh	Stored	Fresh	Stored	Fresh	Stored	Fresh	Stored
Longitude	R	-0.150	-0.105	0.200	0.038	0.052	0.126	0.013	0.264
	P	0.579	0.699	0.459	0.890	0.850	0.643	0.963	0.323
Latitude	R	-0.171	-0.207	0.055	0.044	0.088	0.079	0.165	0.286
	P	0.527	0.441	0.839	0.872	0.745	0.772	0.542	0.282
Altitude	R	-0.144	-0.013	-0.413	-0.154	-0.241	-0.208	-0.038	-0.322
	P	0.594	0.961	0.112	0.569	0.370	0.439	0.888	0.224

Table S4A Correlations between climate and germination percentages of fresh and stored seeds of *Stipa bungeana* from eight natural populations incubated at three temperature regimes.

		Germination (%)					
		10/20 ℃		15/25 ℃		20/30 ℃	
		Fresh	Stored	Fresh	Stored	Fresh	Stored
Mean monthly temperature	R	0.620*	0.434	0.352	0.159	0.244	0.500
	P	**0.018**	0.121	0.217	0.587	0.401	0.069
Monthly total rainfall	R	0.282	-0.139	-0.012	-0.039	-0.207	-0.062
	P	0.328	0.636	0.968	0.894	0.477	0.832

“*” Significant correlation between the two factors at 0.05 level.

Table S4B Correlations between climate and germination percentages of fresh and stored seeds of *Stipa bungeana* from eight natural populations incubated at four water potentials.

		Germination percentage (%)							
		CK		-0.2		-0.4		-0.6	
		Fresh	Stored	Fresh	Stored	Fresh	Stored	Fresh	Stored
Mean monthly temperature	R	0.381	0.340	0.412	0.596*	0.652*	0.384	0.440	0.025
	P	0.179	0.235	0.143	**0.024**	**0.012**	0.175	0.115	0.932
Monthly total rainfall	R	-0.189	-0.028	-0.327	-0.039	-0.154	-0.271	-0.006	-0.337
	P	0.516922	0.923	0.253	0.894	0.599	0.348	0.984	0.239

“*” Significant correlation between the two factors at 0.05 level.
